# Editorial: Update on the Biology, Management, and Treatment of Small Cell Lung Cancer

**DOI:** 10.3389/fonc.2020.01783

**Published:** 2020-11-17

**Authors:** Sonam Puri, Benjamin H. Lok, Natasha Leighl, Jhanelle E. Gray

**Affiliations:** ^1^Division of Medical Oncology, Department of Internal Medicine, Huntsman Cancer Institute, University of Utah, Salt Lake City, UT, United States; ^2^Princess Margaret Cancer Centre, University Health Network, Toronto, ON, Canada; ^3^Moffitt Cancer Center, Tampa, FL, United States

**Keywords:** small cell lung cancer, targeted therapy, immunotherapy, radiation therapy, biomarkers

Small cell lung cancer (SCLC) accounts for around 15% newly diagnosed lung cancer cases in the United States. It is a highly aggressive disease with a poor prognosis and a median overall survival of about 10 months (1). SCLC is frequently diagnosed at an advanced stage and is historically treated as a homogenous disease with a backbone of first-line platinum-etoposide based chemotherapy with or without radiotherapy (2). Surgical resection is only feasible in a small subset of patients with early-stage, node-negative disease. More than 60% of patients respond to first-line chemotherapy. The recent addition of immunotherapy to standard chemotherapy has led to a modest improvement in survival compared to chemotherapy alone for patients with extensive-stage disease (3, 4). Unfortunately, the majority of patients develop disease relapse that is recalcitrant with a poor response to the existing second-line therapy options with an average 2-year survival of <5% (5).

At a molecular level, SCLC is characterized by a uniform loss of RB1 and TP53 tumor suppressor genes and amplification of MYC family of genes (*MYC, MYCL, MYCN*). Recent gene expression profiling of SCLC tumors has enabled the identification of substantial molecular heterogeneity in SCLC tumors with the presence of at least four proposed molecular subtypes (defined by high expression of *ASCL1, NEUROD1, YAP1*, and *POU2F3*) with distinct therapeutic vulnerabilities (6–10). These advances have ushered a new wave of excitement surrounding novel therapeutic targets and biomarkers of response to treatment in SCLC patients.

Contributions to the Research Topic “Update on the Biology, Management and treatment of Small Cell Lung Cancer” highlight the historical data, novel biomarkers under development, and provide a comprehensive review of the recent developments in the understanding of the biology and treatment strategies in patients with SCLC.

The current Research Topic provides some insights on novel biomarkers in development for SCLC. Sone et al. have proposed aberrant expression of nestin (a type VI intermediate filament protein) as a biomarker of decreased sensitivity to chemotherapy and poor prognosis in SCLC. This was based on pre-clinical studies performed by the authors on nestin knock-down cells and nestin overexpressing SCLC cell lines. Additionally, the hypothesis was validated on clinical samples from 84 patients with SCLC, where the authors found that nestin was overexpressed in 28.6% (24/84) patients and was associated with shorter progression-free survival (PFS) following second-line chemotherapy (median PFS 81 days vs. 117 days *P* = 0.029 in nestin-positive vs. nestin-negative patients respectively). In another study, the prognostic and clinic pathological significance of programmed cell death ligand 1 (PD-L1) expression in SCLC was evaluated in a meta-analysis performed by Cai et al.. The final analysis included 921 patients with SCLC from 9 eligible studies and did not identify PD-L1 expression as a prognostic factor (11).

The review by Saltos et al. highlights the evolving role of immunotherapy in the treatment of SCLC, with a comprehensive discussion on the pivotal positive and negative trials, and the differences in trial designs that have shaped the incorporation of immunotherapy in the current management of SCLC. The authors also describe the important areas of ongoing investigation, including the role of immunotherapy in patients with limited-stage SCLC, biomarker development for response to immunotherapy, and additional combinatorial and novel cellular therapy approaches under development.

Taniguchi et al. have eloquently reviewed the topic of targeted therapy in the treatment of SCLC. The article summarizes our current understanding of the various signaling pathways and targets of therapeutic significance in SCLC. The authors have also described emerging therapeutic targets and biomarkers of targeted therapy for SCLC (12).

The review by Tjong et al. focuses on the role of radiation therapy in treatment of SCLC. The authors discuss the data surrounding the different radiation techniques, the evolving role of consolidative thoracic radiation, prophylactic cranial radiation, and stereotactic radiosurgery in the management of SCLC, followed by a brief description on the future of radiation therapy in the era of novel systemic therapies. This is followed by a meta-analysis of studies evaluating efficacy and toxicity of twice-daily vs. once-daily concurrent chemo radiotherapy for patients with limited-stage SCLC written by Wu et al.. The authors evaluated the study level data from 5 randomized control trials meeting the eligibility criteria. The key findings from the analysis showed that twice-daily radiation with concurrent chemotherapy was associated with a higher complete response rate (RR = 1.31, 95%CI 1.01–1.70, *p* = 0.04) and an improvement in overall survival (Hazard ratio, HR = 0.88, 95% CI 1.01–1.12, *p* = 0.03) compared to once-daily radiation, with a similar safety profile within the two groups (13).

Finally, Zhang et al. presented a hypothesis-generating study evaluating surgical resection as a treatment modality for patients with locally advanced stage III SCLC. The authors conducted a retrospective analysis of the Surveillance, Epidemiology, and End Results (SEER) and found 234 patients that underwent surgical resection for stage III SCLC. Compared to the non-surgical group, a higher proportion of stage III treated with surgical resection had either a lower tumor (T1) or a lower nodal (N0/N1) burden, and a lower portion of patients had a high tumor (T4) or nodal (N3) burden. The final survival analysis before and after propensity score matching showed a benefit to surgical resection compared to no surgical resection.

These manuscripts published as part of this Research Topic provide a snapshot of the current treatment paradigm and the ongoing research efforts for our patients with SCLC.

## Author Contributions

All authors listed have made a substantial, direct and intellectual contribution to the work, and approved it for publication.

## Conflict of Interest

The authors declare that the research was conducted in the absence of any commercial or financial relationships that could be construed as a potential conflict of interest.
